# Reversible electroporation for cancer therapy

**DOI:** 10.1093/bjr/tqae231

**Published:** 2024-11-23

**Authors:** Taha Shiwani, Simran Singh Dhesi, Tze Min Wah

**Affiliations:** Department of Diagnostic and Interventional Radiology, St. James’s University Hospital, Leeds Teaching Hospitals NHS Trust, Beckett St, Leeds, LS9 7TF, United Kingdom; Department of Diagnostic and Interventional Radiology, St. James’s University Hospital, Leeds Teaching Hospitals NHS Trust, Beckett St, Leeds, LS9 7TF, United Kingdom; Department of Diagnostic and Interventional Radiology, St. James’s University Hospital, Leeds Teaching Hospitals NHS Trust, Beckett St, Leeds, LS9 7TF, United Kingdom

**Keywords:** reversible, electroporation, electrochemotherapy, electropermeabilisation, electrotransfer, immunotherapy, ablation, intervention, oncology, calcium

## Abstract

Reversible electroporation (EP) refers to the use of high-voltage electrical pulses on tissues to increase cell membrane permeability. It allows targeted delivery of high concentrations of chemotherapeutic agents including cisplatin and bleomycin, a process known as electrochemotherapy (ECT). It can also be used to deliver toxic concentrations of calcium and gene therapies that stimulate an anti-tumour immune response. ECT was validated for palliative treatment of cutaneous tumours. Evidence to date shows a mean objective response rate of ∼80% in these patients. Regression of non-treated lesions has also been demonstrated, theorized to be from an *in situ* vaccination effect. Advances in electrode development have also allowed treatment of deep-seated metastatic lesions and primary tumours, with safety demonstrated *in vivo*. Calcium EP and combination immunotherapy or immunogene electrotransfer is also feasible, but research is limited. Adverse events of ECT are minimal; however, general anaesthesia is often necessary, and improvements in modelling capabilities and electrode design are required to enable sufficient electrical coverage. International collaboration between preclinical researchers, oncologists, and interventionalists is required to identify the most effective combination therapies, to optimize procedural factors, and to expand use, indications and assessment of reversible EP. Registries with standardized data collection methods may facilitate this.

## Introduction

Electroporation (EP) refers to the targeted use of high-voltage electrical pulses on tissues to increase cell membrane permeability. It may be “irreversible” or “reversible”: the former has permanent effects on cellular homeostasis and leads to apoptosis, while the latter causes transient change to allow intracellular migration of large molecules or hydrophilic compounds.^1^ Electrochemotherapy (ECT) refers to the specific use of reversible EP to provide targeted delivery of chemotherapeutic agents to tumours, with clinical efficacy first demonstrated for head and neck cancer in the early 1990s.^2^

ECT is most established in the palliative treatment of cutaneous and subcutaneous tumours. The updated European Standards of Practice for Electrochemotherapy (ESOPE) published in 2018 defined the key indications for ECT, including the treatment of symptomatic cutaneous metastases or refractory primary skin tumours.^3^ However, ECT is increasingly being used for the treatment of deep-seated primary and metastatic solid tumours, with a review in 2023 identifying early-stage studies of ECT on liver, kidney, pancreas, bone, and gastric tumours.^4^ Additionally, reversible EP is now being used to deliver alternative anti-tumoural therapies, with strategies such as calcium EP and gene electrotransfer.^5^

This article provides a narrative review of the theoretical basis, practical considerations, and clinical evidence base of reversible EP and associated anti-tumoural therapies. Information is collated from the most recent and relevant review articles (Table 1) with much of the primary evidence derived from small, early stage-clinical trials, although some larger cohort studies have investigated ECT in cutaneous and certain deep-seated tumours.

**Table 1.** A summary of key reviews on reversible electroporation for cancer therapy.

Review focus	Review title	Year of publication
Electrochemotherapy	**Cutaneous tumours** Electrochemotherapy with intravenous bleomycin for patients with cutaneous malignancies, across tumour histology: a systematic review.^6^	2022
	**Deep-seated tumours** Optimal dosing and patient selection for electrochemotherapy in solid abdominal organ and bone tumors.^4^	2023
	**Liver tumours** Percutaneous electrochemotherapy (ECT) in primary and secondary liver malignancies: a systematic review.^7^	2023
Calcium electroporation	A comprehensive review of calcium electroporation—a novel cancer treatment modality.^8^	2020
Gene electrotransfer and immunotherapy	Electrochemotherapy combined with immunotherapy—a promising potential in the treatment of cancer.^9^	2023
Research directions	Pulsed electric fields in oncology: a snapshot of current clinical practices and research directions from the 4th World Congress of Electroporation.^10^	2023

## Theoretical basis

**Figure 1.**
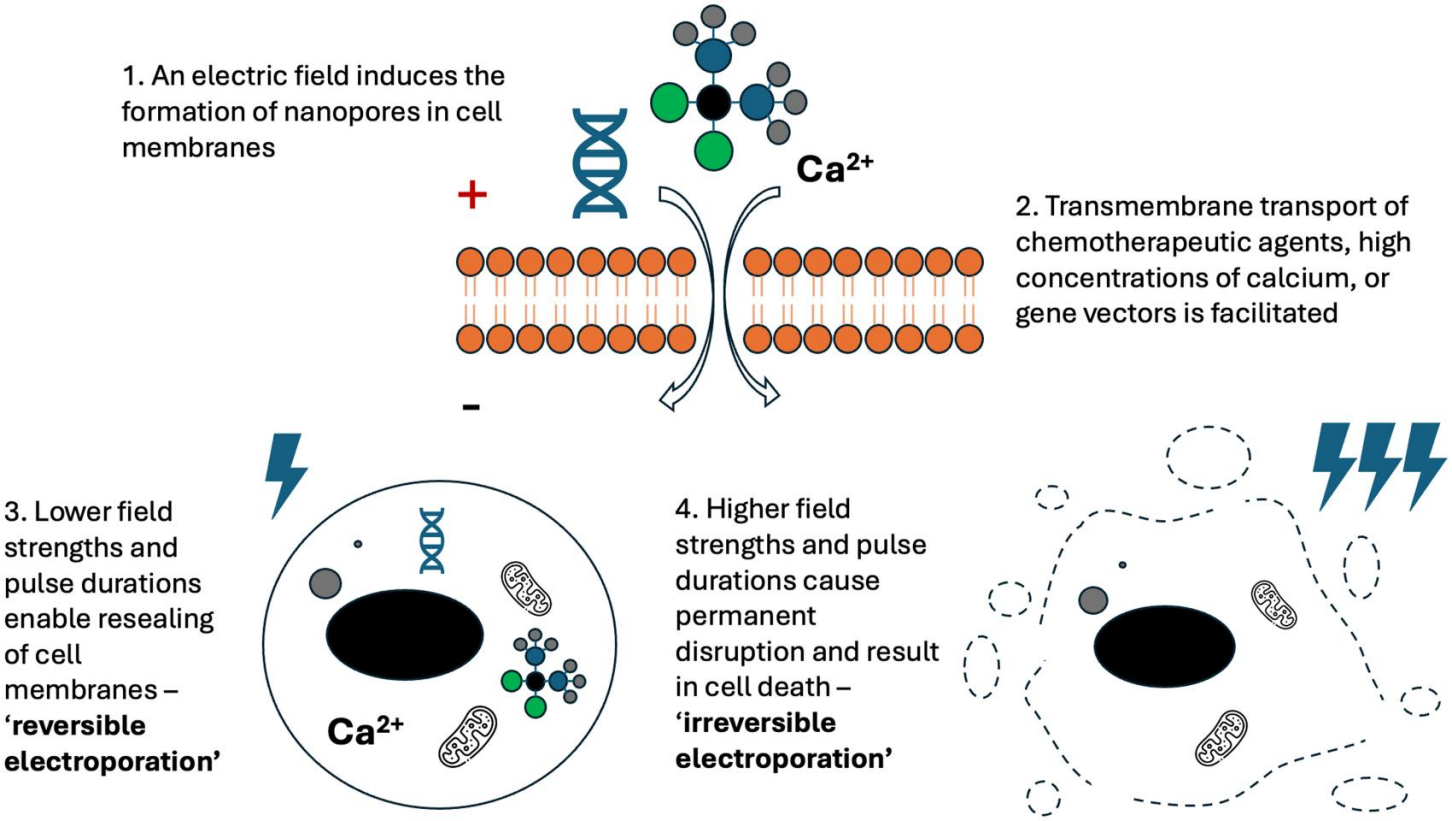
An illustration of the theoretical basis of reversible and irreversible EP. Abbreviation: EP = electroporation.

The prevailing theory of reversible EP is that external electric fields induce a voltage across cell membranes that leads to the incorporation of water molecules within their lipid bilayer and the formation of nanopores. Pore resealing takes several orders of magnitude longer than pore formation, creating a window for transmembrane mass transport, with cancer cells resealing more slowly than normal cells *in vitro*.^11^ If the resealing is too slow, or there is excess mass transport, then cell death can occur (irreversible EP). Higher field strengths and pulse durations increase the likelihood of irreversible effects, and cell size and cell medium can also affect this probability.^12^ Importantly, some effects of EP on cell membranes are mediated by direct effects on membrane lipids and transmembrane proteins, rather than the movement of water molecules.^13^ This process is summarized in Figure 1.

Bleomycin and cisplatin are the only well-established chemotherapeutic agents that demonstrate increased toxicity when delivered by reversible EP.^14^ Both drugs are poorly permeable under normal circumstances but have high intrinsic cytotoxicity and a high therapeutic index when used in ECT—bleomycin toxicity is increased almost 800-fold, while cisplatin toxicity is increased 80-fold.^15^ Bleomycin is most used clinically and may be more useful than cisplatin in radiotherapy resistant tumours^16^ although it may be less effective than cisplatin in cancers associated with human papilloma virus.^17^

**Figure 2.**
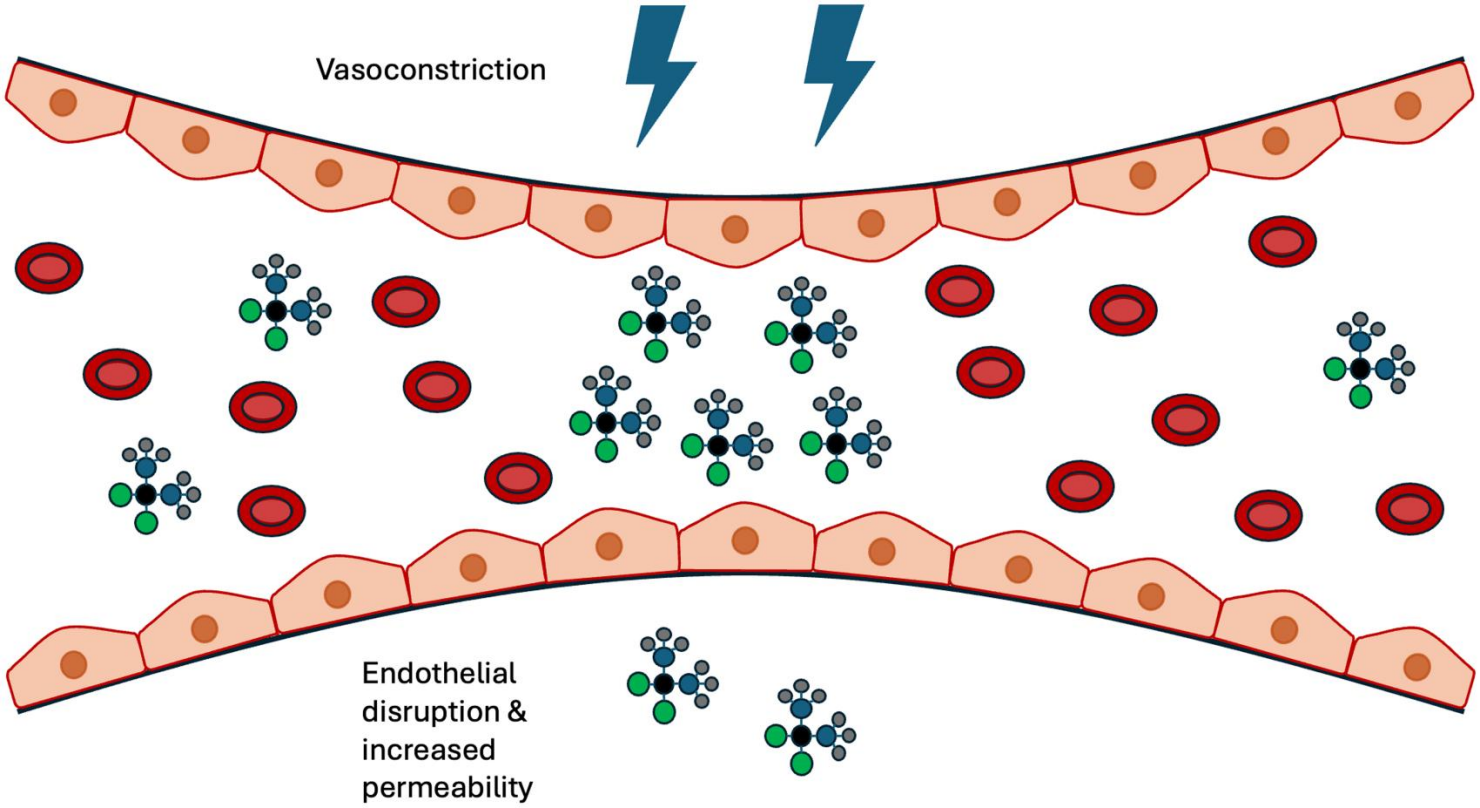
A depiction of the “vascular lock” phenomenon, whereby the electric field applied during ECT causes vasoconstriction that reduces tumour blood flow and decreases drug washout. It also disrupts endothelial cells, increasing delivery of chemotherapeutic agents.^18^ Figure adapted from Jenkins et al^19^ under Creative Commons license BY 4.0. Abbreviation: ECT = electrochemotherapy.

**Figure 3.**
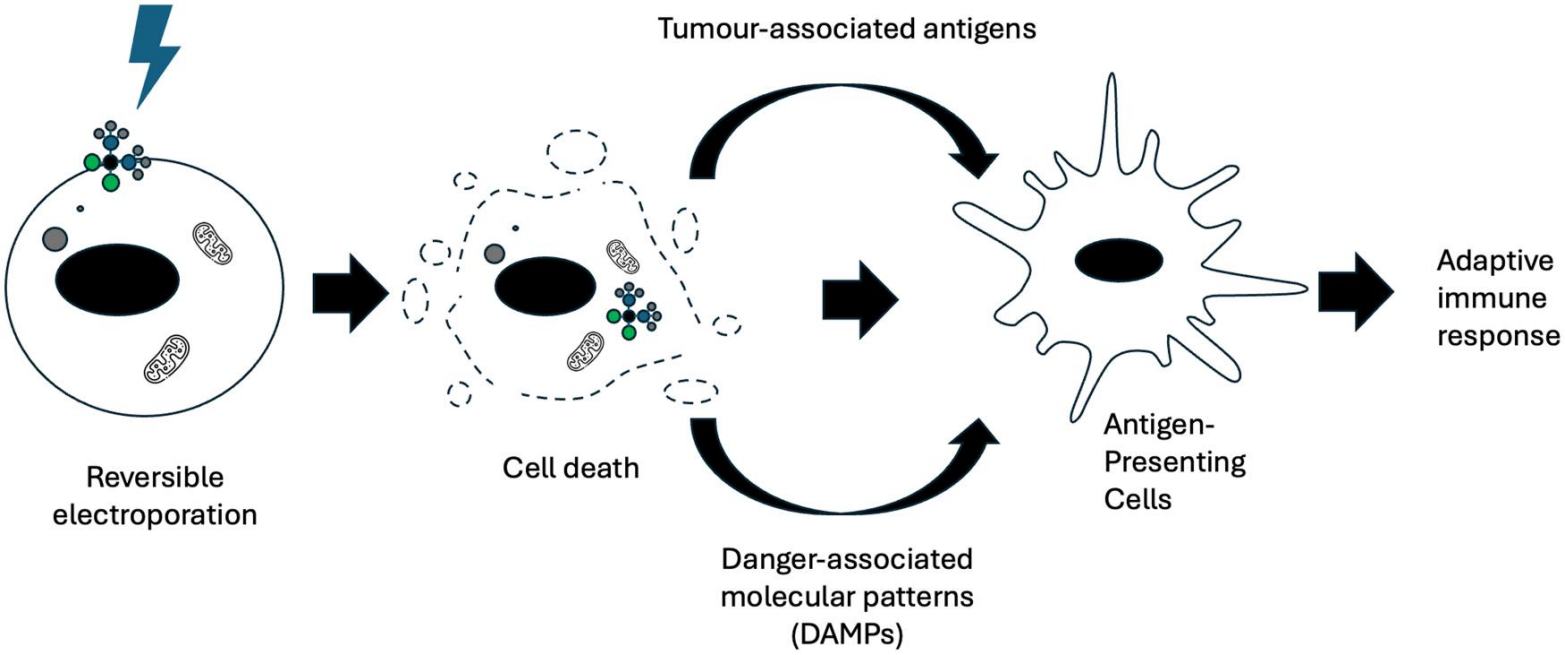
A summary of the process by which ECT establishes an anti-tumoural immune response. Cell death induced by ECT results in the recruitment and activation of antigen-presenting cells through the release of damage-associated molecular patterns and tumour-associated antigens. These cells subsequently stimulate an adaptive anti-tumoural immune response.^20^ Abbreviation: ECT = electrochemotherapy.

Notably, chemotherapeutic toxicity of ECT *in vivo* is greater than that seen *in vitro*, which has been attributed to additional vascular and immune effects of ECT.^18^ Specifically, ECT disrupts tumour vascular endothelial cells and induces vasoconstriction that reduces both tumour blood flow and drug washout, increasing local chemotherapeutic concentration (a “vascular-lock” effect) as shown in Figure 2.^18^ ECT also induces T-cell dependent tumour cell death by stimulating the release of damage-associated molecular patterns and tumour associated antigens, establishing an anti-tumoural immune response as shown in Figure 3.^21^ This may be necessary for treatment success; a study of ECT in mice found that curability was not achieved in immunodeficient mice, with tumour growth delay being half as long compared to immunocompetent mice.^21^ The anti-tumoural immune response may additionally explain abscopal effects—systemic anti-cancer effects following localized treatments—which have occasionally been observed in ECT studies.^22^ These effects have been more extensively documented in localized radiotherapy, where an immune-mediated mechanism is similarly thought to be the underlying cause.^23^

Gene electrotransfer and calcium EP are emerging treatment methods built upon the early evidence of ECT. They respectively refer to the use of reversible EP to deliver gene therapies and toxic concentrations of calcium to tissues. In cancer treatment, gene electrotransfer is most commonly used to develop innate and adaptive anti-tumoural immune responses by delivery of immune-stimulating cytokines (e.g. interleukin-12) and checkpoint-inhibiting antibodies, sometimes in combination with bleomycin or cisplatin.^5^^,^^24^ Conversely, calcium EP induces ATP depletion and selective cancer cell death by leveraging altered calcium homeostasis in cancer cells. It also benefits from similar vascular and immune effects as traditional ECT.^8^^,^^25^ Notably, calcium EP is non-mutagenic, relatively inexpensive, and may have fewer treatment-related adverse events (ulceration, exudation, itching, hyper-pigmentation) compared to treatment with bleomycin.^26^

## Practical considerations

The ESOPE published in 2006 provided the first practical framework for reversible EP therapy, specifically for ECT of cutaneous and subcutaneous tumours smaller than 3 cm.^27^ It provided numerous practical protocols, all of which involved administration of bleomycin or cisplatin followed by a series of electrical pulses (8 pulses at 100 microseconds each) delivered by electrodes inserted into the tumour. Plate or needle row electrodes were recommended for smaller, superficial nodules, and hexagonal needle electrodes were recommended for larger nodules due to better coverage (albeit a higher risk of hyper-pigmentation). General anaesthetic was preferred over local anaesthetic if multiple nodules or nodules larger than 0.8 cm were being treated. As the evidence base for ECT expanded, the ESOPE guidelines were updated in 2018 to include guidance for superficial tumours larger than 3 cm.^3^

The guidelines do not yet include the use of variable electrode geometry electrodes, which allow the treatment of deep-seated tumours through adjustable axial displacement.^28^ These treatments are usually performed under image guidance and require both modelling and individualized treatment planning to determine the electrode positions that provide the required electrical field coverage and distribution.^29^ Newer pulse generators also allow the electrical field to be varied according to tumour size and geometry.^30^ Unfortunately, variations in electrical conductivity in different tissues and in regions affected by necrosis, vasculature, and micro-heterogeneities can limit the reliability of numerical models, reducing the accuracy of electrode positioning and subsequent clinical success of treatment.^31^

Compared to other minimally invasive oncological treatments such as radiofrequency ablation, ECT is a non-thermal technique that does not damage surrounding organs or vessels.^28^ However, ECT procedures can be of longer duration, limiting utility for multifocal tumours. Additionally, side effects from the chemotherapeutic agents used in ECT (eg, bleomycin-induced pulmonary fibrosis) may occur despite use of relatively small doses, and there are theoretical risks of arrhythmia induction if ECT is performed close to the heart, although this may be mitigated by electrocardiogram synchronization.^32^

Research to optimize ECT protocols is ongoing. Recent studies favour nanosecond electrical pulses over microsecond pulses due to proposed advantages including reduced muscle contraction during treatment delivery, less thermal damage to tissues, and less electrolysis of electrodes.^33^ Moreover, ECT success likely depends upon histological tumour type^34^ which does not yet factor in ECT protocols. Trials have also demonstrated the feasibility of lowering the bleomycin dose currently used in ECT to reduce the risk of side effects, particularly in older patients or those with impaired renal or respiratory function.^6^

## Evidence base

**Figure 4.**
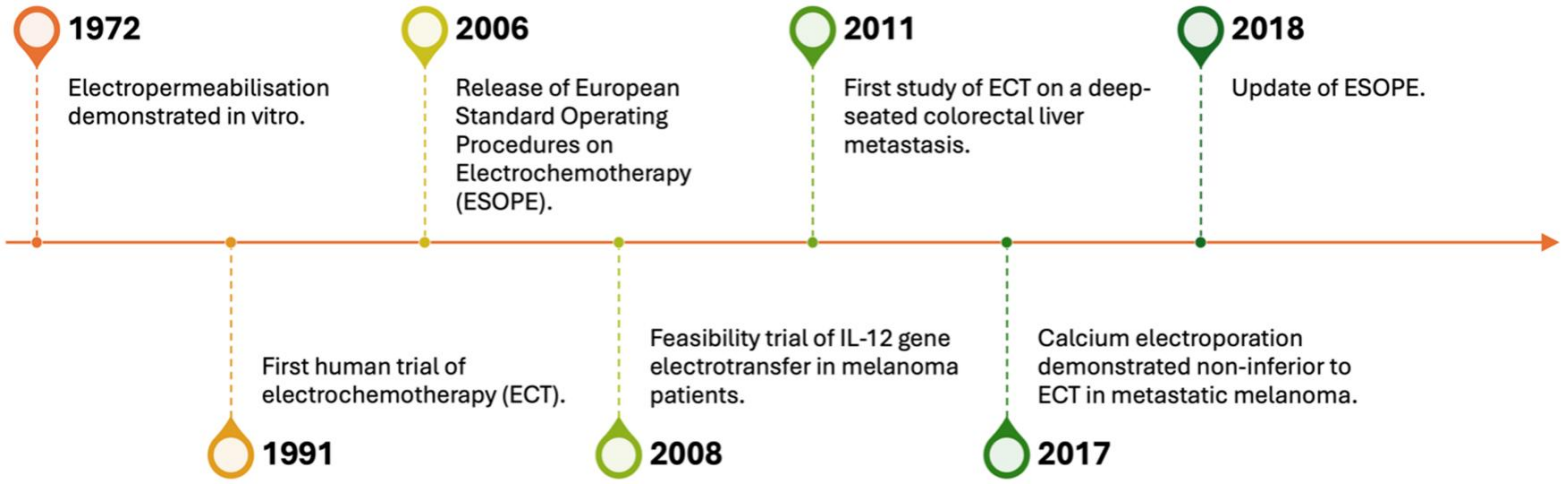
A timeline of research on reversible electroporation for cancer therapy.

Figure 4 provides an approximate timeline of the evidence base around reversible EP for cancer therapy. Neuman and Rosenheck^35^ first demonstrated a transient increase in cell membrane permeability following electrical pulse administration *in vitro* in 1972. Mir et al^2^ then performed the first clinical trial of ECT in 8 patients with head and neck squamous cell cancer in 1991, treating 40 nodules with a complete clinical response rate of 57%. Numerous studies have since evaluated the effectiveness of ECT for: cutaneous and subcutaneous tumours, deep-seated metastatic malignancies, and deep-seated primary tumours. Studies have also evaluated calcium EP, immunotherapy, and gene electrotransfer for cancer therapy. The evidence base of these treatments has been evaluated below, with a focus on human trials.

### Cutaneous and subcutaneous metastasis

Following the first trial by Mir et al,^2^ several early-stage clinical trials evaluated the feasibility and optimal dosage of ECT for various cutaneous and subcutaneous tumours, primarily malignant melanoma. This culminated in the publication of the first ESOPE guidelines in 2006 which standardized treatment protocols.^36^ The protocols were validated with a prospective study on 41 patients with progressive cutaneous and subcutaneous metastases. Results showed an objective response rate of 85% (at least partial tumour reduction) and a complete response rate of 74% (total tumour eradication). Minimal adverse events were reported.^36^

Subsequent studies corroborated those results. A systematic review published in 2022 identified 55 studies evaluating ECT with intravenous bleomycin on patients with cutaneous malignancies.^6^ The mean objective response rate in all 3729 patients was determined to be 81.9%. Only 1 patient had a serious adverse event related to ECT (sepsis post-treatment in a patient with a large ulcerated tumour^37^) the only other adverse events described included mild pain and skin hyperpigmentation. A significant proportion of studies included in this review were part of the influential International Network for Sharing Practice in ECT (INSPECT) pan-European Collaborative which prospectively collected data on the use of ECT for cutaneous malignancy over 11 years.^38^

Importantly, these studies highlighted numerous factors that influence response rate, including tumour size, histological type, number of lesions, previous radiotherapy, and number of electrode applications and ECT cycles. In particular, larger tumours which had been previously irradiated showed reduced responses to ECT, theorized to be due to disrupted vasculature in irradiated tissues reducing chemotherapeutic uptake and fibrotic skin reducing current delivery.^6^

Questions remain regarding the impact of ECT on quality of life which is of particular importance in treatments with palliative intent. One study which assessed quality of life in 378 patients with melanoma treated with ECT found no significant change following treatment.^39^ Additionally, there is minimal data directly comparing the effectiveness of ECT to alternative palliative treatments for cutaneous and subcutaneous tumours such as radiotherapy, isolated limb perfusion, intralesional therapies, or systemic chemotherapy. Notably, ECT only requires a single treatment session and is relatively cost- and time-efficient,^40^ however, multifocal tumour ECT treatments may necessitate prolonged treatment times compared to other ablative treatments.^10^

### Deep-seated tumours

Since the first study investigating the use of ECT on colorectal liver metastases in 2011,^41^ the safety and feasibility of ECT for a variety of deep-seated tumours (primarily palliative or treatment-refractory tumours) has been investigated extensively, as summarized in a recent systematic review.^10^

Importantly, challenges of ECT described previously are more pronounced in the treatment of deep-seated tumours, with conductivity variations, electrode positioning, cardiac synchronization, and the need for general anaesthesia being particularly relevant.^42^^,^^43^ Some of the key evidence has been highlighted below, with unique factors for intra-abdominal tumours, bone tumours, and endoluminal treatments.

#### Intra-abdominal tumours

Intra-abdominally, ECT has been used most extensively in the treatment of metastatic and locally advanced hepatic malignancy,^4^ with evidence highlighting a good safety profile despite complex anatomy and frequent associations with parenchymal disease.^43^^,^^44^ Indeed, studies have investigated both percutaneous and intraoperative treatment, and shown feasibility of ECT for hepatocellular carcinoma, cholangiocarcinoma, colorectal liver metastases, and other metastases.^10^ A 2023 systematic review identified 8 studies investigating the effect of ECT on primary and secondary liver malignancies, the largest of which included treatment of 39 liver tumours.^7^ Reported complete response rates varied widely from 55% to 100%. The review highlighted the absence of risk to large vessels (>5 mm) and bile ducts after ECT treatment, reinforcing the evidence from animal studies that ECT does not affect the function or architecture of larger tumour vessels or healthy liver parenchyma.^45^ This contrasts with existing thermal and non-thermal ablation techniques with which ECT has comparable effectiveness.^7^ While transarterial chemoembolization may be used in similar patient cohorts and allows locally controlled chemotherapy, it does not enhance chemotherapeutic drug uptake.^43^

Pancreatic adenocarcinoma and renal cell carcinoma have also been investigated, but on a much smaller scale. Two studies investigated the feasibility of ECT for locally advanced pancreatic adenocarcinoma using an open surgical approach and reported no serious adverse events.^46^^,^^47^ A few small studies trialled ECT for stage 3 advanced and stage 4 metastatic pancreatic adenocarcinoma, but response rates were variable, although results suggested improvements in quality of life.^48–51^ ECT of stage 3 renal cell carcinoma has been described separately in 2 individual case reports with complete response on post-treatment CT imaging; however, treatment was not standardized.^52^^,^^53^ There remains little evidence for ECT in these tumours, and further research is required before clinical adoption.

#### Bone tumours

Bone tumours are relatively rare and can be difficult to treat with ECT if located in the vertebrae due to the need for the active tips of electrodes to be parallel to each other.^54^ However, ECT of bone metastases is of particular interest because of their associated pain, either from nerve compression, fractures, or impaired mobility.^10^ One study of ECT in 38 patients with bone metastases demonstrated an objective response rate in 29% and disease stabilization in 59% of subjects.^54^ Another study found a significant decrease in pain in 23/29 subjects with bone metastasis, with a median visual numeric scale pain control score reduction of 2.3.^55^ Unlike other treatments, ECT does not cause bone necrosis and therefore preserves bone architecture, mechanical stability and healing capability in case of fracture or need for surgical intervention.^54^ However, it cannot be performed if any metal implants are in place.

#### Endoluminal ECT

Endoscopic ECT is being explored for oesophageal and colorectal cancer, with phase 1 studies in 2018 and 2020 showing promise.^56^^,^^57^ Both used the EndoVE^®^ device (Mirai Medical, Galway, Ireland) and demonstrated feasibility and safety of treatment, although there were issues with tumour coverage. The device used consisted of electrodes attached to an endoscope—tumour tissue was brought into contact with the electrodes by use of a vacuum. Newer devices such as the Stinger^®^ device (IGEA S.p.A., Modena, Italy) have since been developed and investigated.^58^ If electrode positioning can be optimized for complete tumour coverage, endoluminal ECT may hold significant promise.

### Calcium electroporation

Calcium EP was first tested in humans in 2017 in a phase 2 study of 7 patients with a total of 47 cutaneous metastatic lesions.^26^ Metastases were randomized to receive calcium EP or ECT with intratumoural bleomycin in a test of non-inferiority, with a threshold of a 15% difference in response rates. No statistically significant difference was found between the treatments with objective response rates for calcium EP and ECT being 72% and 84%, respectively. Both treatments were less successful in tumours that had previously been irradiated, and calcium EP was associated with less adverse hyperpigmentation than ECT. Abscopal effects were also noted in one patient with malignant melanoma who received both ECT and calcium EP for metastatic lesions, with complete remission of malignancy 9 months after treatment.

Non-inferiority of calcium EP to ECT was confirmed in another phase 2 trial with 7 patients in 2020 (with a threshold of 20% response rate difference)^59^ and subsequent studies have demonstrated the feasibility of calcium EP in the treatment of advanced oesophageal cancer,^60^ colorectal cancer,^61^ recurrent head and neck cancer,^62^ and cutaneous manifestations of gynaecological cancers^63^ albeit with mixed tumour responses. A larger-scale superiority trial comparing calcium EP against ECT is required to determine the precise utility of calcium EP. If suitably effective, it may provide a viable alternative to ECT and may be associated with fewer cosmetic side effects, lower costs, and less storage/handling difficulty.

### Gene electrotransfer and immunotherapy

In 2008, Daud et al^64^ tested gene electrotransfer of an immune-stimulating IL-12 plasmid in a phase 1 trial of 24 patients with malignant melanoma, which otherwise had significant toxicity concerns when delivered systemically. Two patients demonstrated complete cancer regression (including non-treated lesions), while another 8 patients had a partial response to therapy. Transient pain was the only recorded adverse event. A marked CD4+ CD8+ infiltrate was demonstrated on biopsy, indicative of anti-tumoural immune response generation.

A subsequent phase 2 trial in patients with melanoma^65^ and other trials of IL-12 EP for the treatment of Merkel cell carcinoma^66^ and triple-negative breast tumours^67^ confirmed safety of treatment with partial tumour responses. The latter trial highlighted how IL-12 increases tumour antigen presentation and T-cell response, and also sensitizes cells to treatment with anti-PD-1/anti-PD-L1 immune checkpoint inhibitors. This was demonstrated clinically in the same study; one patient who had previously been resistant to anti-PD-1/anti-PD-L1 therapy demonstrated response to this therapy following IL-12 EP. Combination treatment (anti-PD-1/anti-PD-L1 therapy and IL-12 gene electrotransfer) was therefore directly tested in a phase 2 trial of patients with metastatic melanoma.^68^ Combined IL-12 and anti-PD-1 therapy significantly increased tumour immune infiltration and resulted in an anti-tumoural immune response with an objective response rate of 41%.

Immunotherapies have been also tested with ECT, independent from gene electrotransfer. ECT is thought to generate an anti-tumoural immune response which has been dubbed a form of “*in situ* vaccination”, and may be enhanced by checkpoint inhibitors (anti-CTLA4 or anti-PD-1).^69^ A 2023 review identified 7 studies which had investigated such effects on cancers including melanoma, breast cancer, hepatocellular carcinoma, and squamous cell carcinoma.^9^ Studies on melanoma and breast cancer that compared effectiveness of ECT combined with immunotherapy demonstrated increased efficacy of ECT with immunotherapy compared to either therapy alone. However, the studies included small numbers of participants and were not randomized.

This area remains a very active field of research, with pre-clinical studies investigating gene electrotransfer of other cytokines such as TNFα and IL-2^5^ and combination therapy of calcium EP and cytokine gene electrotransfer.^70^ Gene electrotransfer has also been investigated as a means for the delivery of cancer-specific antigens as a form of DNA vaccine, particularly for urological cancers.^71^

## Conclusions and future directions

The National Institute for Health and Care Excellence (NICE) in the United Kingdom approved the use of ECT for the palliative treatment of cutaneous and subcutaneous metastases in 2013.^72^ Evidence since then suggests that reversible EP cancer therapies are a safe treatment option even for primary deep-seated tumours and may be particularly effective when combined with immunotherapies and gene electrotransfer to take advantage of the abscopal effect. A greater understanding of the most effective combination therapies and protocols for different tumour histotypes may enable use of reversible EP as an adjuvant or neoadjuvant tumour therapy outside of the palliative setting, particularly if biomarkers can identify those most likely to respond to treatment. This may facilitate individualized treatments whereby combination reversible EP therapies are personalized to a patient’s tumour factors and likelihood of response. In 2021, Falk et al developed a research roadmap highlighting potential biological factors that may predict response to ECT, including specific tumour cell characteristics and tumour microenvironment factors.^69^

There remains scope to optimize reversible EP protocols and techniques. Use of nanosecond electrical pulses, calcium EP and lower bleomycin doses in ECT may reduce the rare adverse events associated with reversible EP therapies. Conscious sedation may provide a feasible and more accessible alternative to general anaesthesia in the treatment of multiple/larger tumours in specific cases, as demonstrated by a recent small prospective study.^73^  *In vivo* experiments are also ongoing to improve models of electrical field distribution in heterogenous tissues, such as a 2023 study which developed a model that can account for temperature variation and non-parallel electrodes.^74^ It may even be possible to monitor and adjust EP therapies in real-time using techniques such as rapid impedance spectroscopy.^75^ Many of these key research directions were highlighted and categorized following the World Congress on Electroporation in 2022, with the subsequent collaborative publication providing a good summary of the limitations of evidence to date.^10^

International collaboration between medical device developers, preclinical scientists, oncologists and interventional radiologists is needed to focus efforts on the most promising treatment protocols and combination therapies in the field, particularly looking at the treatment of deep-seated tumours, where reversible EP has significant advantages over current minimally invasive treatment methods. Increased awareness of reversible EP therapies among oncologists and referring clinicians will also increase the volume of reversible EP cases available for analysis. Ideally, cases should be recorded in an international registry with standardized data collection and reporting methods, like the previously described InspECT registry.^39^ This will enable validation of proposed protocols through large-scale observational studies with reduced heterogeneity, which may in turn justify comparative trials that evaluate reversible EP therapies against existing palliative and adjuvant or neoadjuvant therapies. A multicentre European observational study has already been established to assess the effectiveness of percutaneous ECT for primary or secondary liver cancer, with data being collected in the RESPECT (REgiStry for Percutaneous ElectroChemoTherapy) registry.^76^ The primary endpoint is to assess local tumour control 12 months after treatment, with a view to including 250 patients. This collaboration is an important first step in providing broader validation for reversible EP therapies.
